# Marginal Strength of Collarless Metal Ceramic Crown

**DOI:** 10.1155/2010/521470

**Published:** 2010-06-01

**Authors:** Sikka Swati, R. Chowdhary, P. S. Patil

**Affiliations:** ^1^Department of Maxillofacial Prosthodontics and Implantology, AIIMS Dental College and Hospital, New Delhi 110012, India; ^2^Department of Maxillofacial Prosthodontics and Implantology, HKE'S S. Nijalingappa Institute of Dental Sciences & Research Centre, Gulbarga 585104, India

## Abstract

Metal ceramic restorations have been implicated for the discoloration in area of labiogingivalmargin. Attempts to rectify this, by altering the design of metal frameworkswill lead to decrease in fracture strength atmargin. This in vitro study compared the fracture strength at margins of metal ceramic crowns cemented to metal tooth analogs. Crowns evaluated with different marginal configurations, shoulder and shoulder bevel with 0 mm, 0.5 mm, 1 mm, and 1.5 mm, were selected. *Methods*. Maxillary right canine typhodont tooth was prepared to receive a metal ceramic crown with shoulder margin. This was duplicated to get 20 metal teeth analogs. Then the same tooth was reprepared to get shoulder bevel configuration. These crowns were then cemented onmetal teeth analogs and tested for fracture strength atmargin on an Instron testing machine. A progressive compressive load was applied using 6.3 mm diameter rod with crosshead speed of 2.5 mm per minute. Statisticaly analysis was performed with ANOVA, Student's “*t*” test and “*f*” test. *Results*. The fracture strength of collarless metal ceramic crowns under study exceeded the normal biting force. Therefore it can be suggested that collarless metal ceramic crowns with shoulder or shoulder bevel margins up to 1.5 mm framework reduction may be indicated for anteriormetal ceramic restorations. *Significance*. k Collarless metal ceramic crowns have proved to be successful for anterior fixed restorations. Hence, it may be subjected to more clinical trials.

## 1. Introduction

Metal ceramic crown has always been the most popular complete veneer restoration in dentistry, because it derives its aesthetics from the highly translucent natural appearance of porcelain and the strength from the metal substructure [1, 2]. But optimum aesthetics is not achieved consistently with conventional ceramometal restorations, particularly in area of labiogingival margin [3]. 

Research has shown that metal collars diminish light transmission into the adjacent tooth tissue, causing darkened appearance of both root surfaces and gingival [4]. Hence, the choice of restoration would be, that has the structural advantages of the metal ceramic restoration and the aesthetic qualities of the all ceramic crown, especially on the cervical third [5]. These requirements have led to the development of facial porcelain margin in metal ceramic crowns, also known as collarless metal ceramic crown.

In collarless metal ceramic crowns, the facial porcelain margin eliminates the unpleasing metal collar, due to increased thickness of porcelain at the gingival margin. Plaque retention is reduced due to highly glazed body porcelain at the margin. As it is not necessary to hide a metal collar, periodontal health is further promoted by minimal extension into the gingival sulcus [6].

Various studies have been done on collarless metal ceramic crowns with different marginal configurations and different framework reduction, which have been checked for fracture strength under vertical load [3, 5]. 

However, occlusal forces acting on the anterior teeth are not generated exactly at 90-degrees, but at an angle [2]. So to check for the durability of these collarless metal ceramic crowns in vivo, we need to evaluate these restorations under the load at particular angle, which the tooth encounters in oral cavity.

This study was planned to compare the collarless metal ceramic crowns with two different marginal configurations; shoulder and shoulder with bevel. The crowns were also compared with different framework reductions of metal coping (0 mm, 0.5 mm, 1 mm, and 1.5 mm), at the labial margin for both configurations. These comparisons were carried on a typhodont tooth, right maxillary canine, by generating the occlusal forces at an angle.

## 2. Materials and Methods

**Figure 1:**
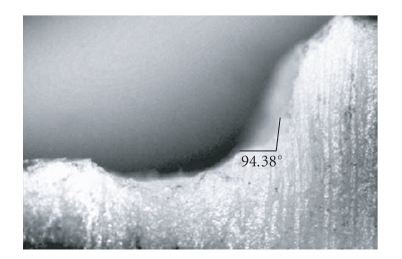
Image on profile projector for Group A.

Biomechanical preparation of a maxillary right canine on the typhodont was done to receive a metal ceramic crown, using an air motor hand piece using diamond burs. Maxillary right canine typhodont tooth was reduced with the required dimension for a metal ceramic crown with 1.5 mm shoulder on the facial surface with 90-degree cavosurface angle. The shoulder on the facial surface was carried to the mid-proximal region both mesially and distally and was blended to a chamfer finish line on lingual surface. The finish line preparation on the facial surface was refined to get shoulder of 1.5 mm with a 90-degree cavosurface angle. Then the finish line angle and dimension were measured with the help of profile projector (Figure 1). A mold of the prepared tooth was made with polyvinylsiloxane putty impression material (Reprosil, Dentsply, USA). Twenty wax patterns were made on this mold, invested in Phosphate bonded investment material (Begavest, Bego, Germany) and casted in nickel-chromium alloy (IPS alloy, Ivoclar Vivadent) with composition of Cr-21-71%, Mo-8-28%, Mn-0.33%, Nb-0.83%, and Ni-balance, to get to 20 teeth analogs.

**Figure 2:**
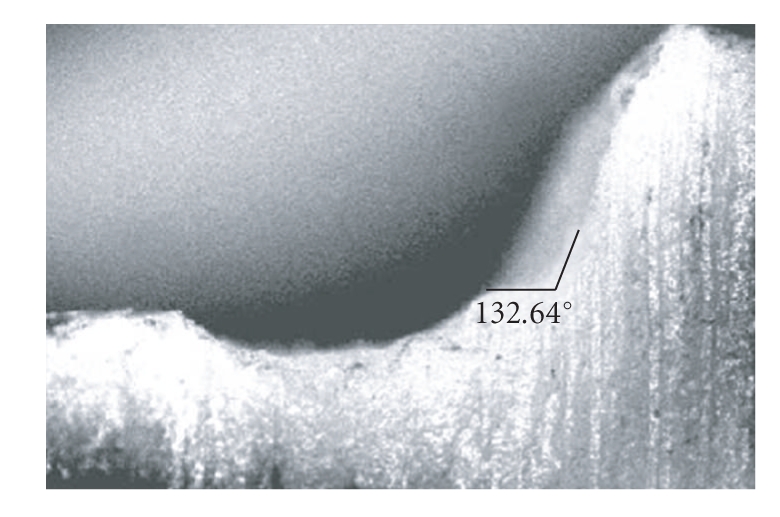
Image on profile projector for group B.

Then maxillary right cuspid tooth (as used in group A) was further reprepared to get a beveled shoulder and short bevel of 0.5 mm dimension with 135-degree cavosurface angle. The preparation was done by mounting the typhodont jaw on the milling machine to give a short bevel of 0.5 mm dimension with 135-degree cavosurface angle. The angle and dimension of the finish line were measured with the profile projector (Figure 2). The mold of this tooth was prepared in the same manner to get 20 metal teeth analogs. Wax patterns for both group A and B were prepared and invested in a phosphate bonded investment material (Begovest, Bego, Germany). The castings were done in the same nickel-chromium alloy. The casting irregularities were removed with rotary instruments and air abrading with 50 *μ*m aluminum oxide with the help of a sandblasting machine.

The metal copings obtained were finished and trimmed on the labial aspect with definite distances from the cavosurface margin. On lingual surface, the metal coping ends at the cavosurface margin for both groups.

Groups A and B were further subdivided into 4 subgroups each: A1, A2, A3, and A4; B1, B2, B3, and B4, respectively.

 Groups A1 and B1: Metal coping extends to cavosurface angle facially.
 Groups A2 and B2: Metal coping is 0.5 mm coronal to cavosurface angle facially.
 Groups A3 and B3: Metal coping is 1 mm coronal to cavosurface angle facially.
 Groups A4 and B4: Metal coping is 1.5 mm coronal to cavosurface angle facially.

The above stated measurements were done using digital Vernier calliper (Camilin, India).

Veneered surfaces of the coping were finished with abrasive wheel to obtain uniform thickness of 0.4 mm, and the castings were cleaned with a 50 *μ*m aluminum oxide air abrasive. Porcelain (IPS Design, Ivoclar Vivadent, Germany) build up was done for both groups A and B using the direct lift off technique. The porcelain build up for both groups of castings were initiated with two applications of opaque porcelain and fired consequently. Shoulder (marginal) porcelain was applied to the groups of castings with porcelain facial margins, by using the direct lift off technique. Shoulder porcelain was brushed to the gingival margins. Then, it was carved with a concavity designed to eliminate the over contouring of the final restoration. This layer was dried and fired. A second corrective layer of shoulder porcelain was applied and fired. Then, dentinal porcelain was applied over the opaque and shoulder porcelain for the crowns with porcelain facial margins. Dentinal porcelain was also applied over the opaque porcelain for the crowns with metal collar margins and was fired.

**Figure 3:**
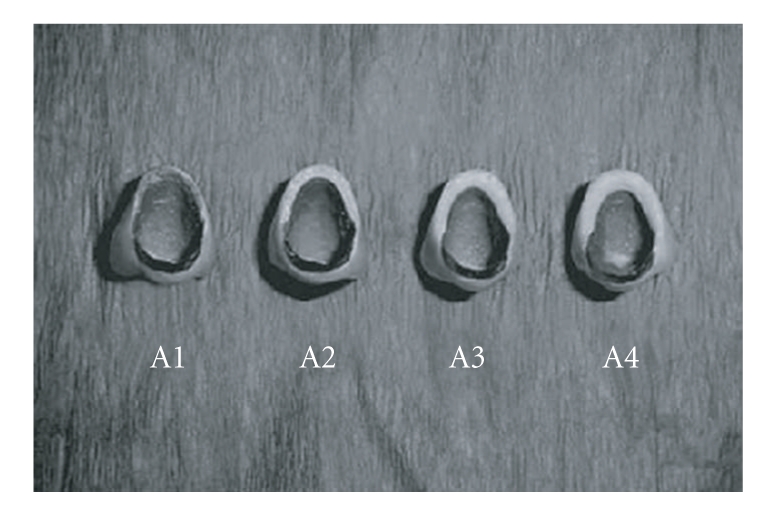
After glazing group A top view.

**Figure 4:**
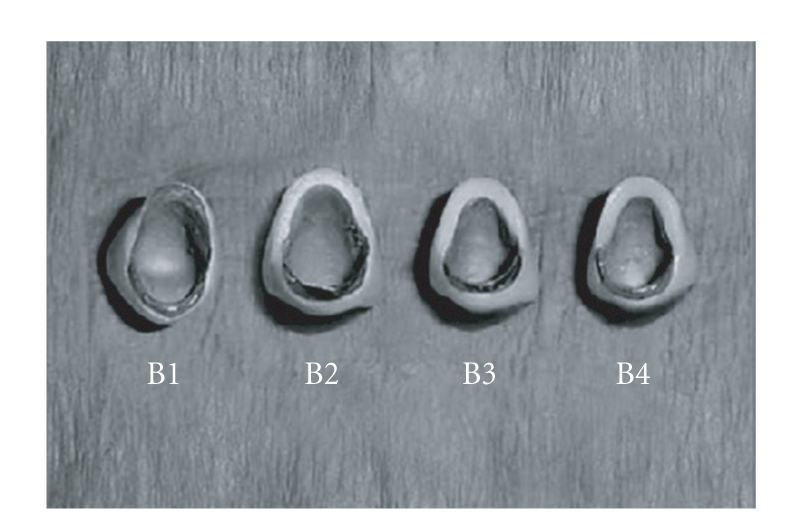
After glazing group B top view.

Incisal porcelain for both the groups was applied in layers and was fired. Finally all the crowns were glazed (Figures 3 and 4).

The crowns were contoured with the abrasive wheels. Measurements were made with a digital Vernier calliper to ensure that the total thickness of porcelain and metal was uniform of 1.5 mm. Then, the porcelain was glazed.

The internal surface of the castings and the surface of the metal analogs were air abraded with 50 *μ*m aluminum oxide. Finished crowns were then cemented with a glass ionomer luting cement Type I (GC, Singapore) to the tooth analogs with a 15 kg static load and allowed to set for 24 hours [1]. The crowned specimens were embedded in an autopolymerizing polymethyl methacrylate resin blocks (DPI, India). The acrylic resin was within 2 mm of the margins of the crowns. All the polymethyl methacrylate resin blocks were ground flat to ensure that each specimen would be secured, and in correct alignment when compressive forces were applied.

**Figure 5:**
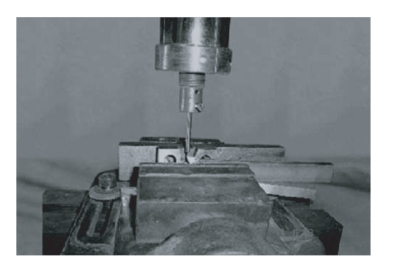
Model on Instron testing machine.

**Figure 6:**
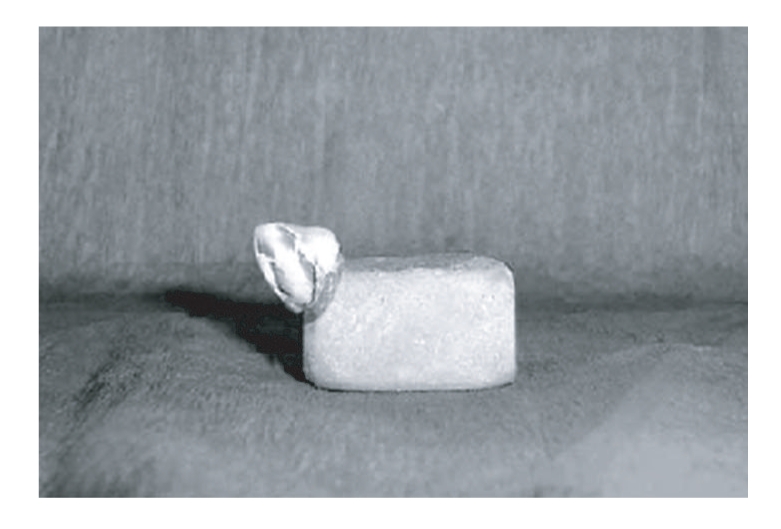
Fractured sample.

Specimens were tested on an Instron testing machine (Kirloskar, India) (Figure 5). The load was directed at linguo-incisal line angle, at 130 degrees to the long axis of the specimen until the catastrophic porcelain fractured (Figure 6). This position was selected to reproduce the occlusal forces directed to a maxillary canine [1]. A 6.35 mm (one quarter inch) diameter rod was used to load the artificial crowns, with the centre of the rod in contact with the porcelain surfaces. A crosshead speed of 2.5 mm per minute was used [1]. Statistical analysis was performed with ANOVA, Student's “*t*” test and “*f*” test.

## 3. Results

**Figure 7:**
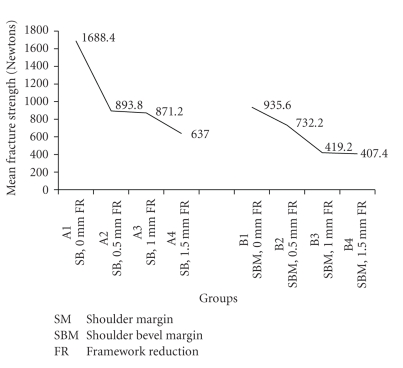
Comparison between mean fracture strength of A and B groups.

Fracture test at margin showed remarkably similar failure modes between the groups, with nearly all specimens failing through a shear fracture of porcelain from load point to facial margin of the crowns. The mean values of the fracture strength at the margin and standard errors of the mean in Newton (N) and comparison between various groups was observed (Table 1 and Figure 7).

### 3.1. Comparison of Fracture Strength with Same Marginal Configuration and Different Framework Reduction

The comparison of fracture strength within group A and within group B was studied by Student's “*t*” test at 5% level of significance. The “*t*” table value for group A was 2.306. It was found that the difference in the fracture strength between A_2_ and A_3_ was statistically insignificant (“*t*” cal 0.3888 < “*t*” tab 2.306). Also, no statistical difference exists between B_3_ and B_4_ (“*t*” cal 0.3153 < “*t*” tab 2.306).

### 3.2. Comparison of Fracture Strength with Different Marginal Configuration and Same Framework Reduction

It was being studied by “*f*” test at 5% level of significance and ANOVA test. Statistically significant difference was found between A and B groups. Also, the difference in the fracture strength for shoulder and shoulder bevel margins with 0 mm and 1 mm framework reductions was much higher than with 0.5 mm and 1.5 mm frame work reductions.

## 4. Discussion

Introduction of porcelain fused to metal restorations was a breakthrough in aesthetically treating dental fixed prosthesis, and specifically improved the aesthetics by eliminating the labial metal collar substitution with porcelain [6]. 

This present study was carried out to test the porcelain marginal failure versus metal collar marginal failure in metal ceramic crowns. In this study, an analog which is similar in size and shape of a human tooth was chosen rather than a regular geometric configuration. Routine dental laboratory waxing, casting, metal finishing, and porcelain application techniques were also selected to mimic clinical procedures. Every effort was made to maintain uniformity in the samples. Several techniques have been developed over the years for fabricating metal ceramic crowns with porcelain facial margin. The best known techniques are Platinum foil technique [5], Refractory die technique [7], Direct lift-off technique [8], and Porcelain wax technique [9]. Out of the various techniques available for fabricating the collarless metal ceramic crowns, direct lift-off technique was proved to be the simplest and easiest [10], and thus used in this study.

Various studies have been done on collarless metal ceramic crowns with different marginal configurations and different framework reductions [1, 2, 4, 11]. But most of them explain the fracture strength on vertical load. However, the occlusal forces acting on the anterior teeth are not generated exactly at 90-degrees, but at an angle [1]. So to check for the durability of these collarless metal ceramic crowns in vivo, we need to evaluate these restorations under load at a particular angle which the tooth encounters in oral cavity. In this study, to test the fracture strength, a progressive compressive load with crosshead speed of 2.5 mm per minute was used, in accordance with the previous studies [1].This was done to allow time for distribution of the forces from the point of application to the porcelain throughout. To test the fracture strength, the load was directed at linguo-incisal line angle, at 130 degrees to the long axis of specimen rather than a vertical load. The marginal configuration has been studied with different framework reduction for collarless metal ceramic crowns. O'Boyle et al. advised 1 mm of facial metal reductions for anterior metal ceramic crowns, according to them, there was a drastic improvement in aesthetics with 1 mm of the metal reduction, without any significant decrease in the fracture strength [4]. Ulusoy and Toksavul evaluated the fracture resistance of metal ceramic restorations with different metal framework reduction. They concluded that as the amount of metal reduction increases, the vertical fracture resistance decreases [2]. However, Gardner et al. found that the fracture strength of porcelain facial margin was significantly higher than porcelain fused to metal margin [1]. Hence, this study was planned to compare the collarless material ceramic crowns with two different marginal configurations, shoulder and shoulder with bevel. The crowns were also being compared with different framework reductions of metal coping: 0 mm, 0.5 mm, 1 mm, and 1.5 mm at the labial margin for the both configurations.

The shoulder marginal configuration with different framework reduction was evaluated. It was found that the fracture strength of crowns with 0.5 mm and 1 mm metal framework reductions for shoulder margin group is nearly the same. Also, the fracture strength of collarless metal ceramic crowns with shoulder margin exceeded the normal masticatory loads. However, the fracture strength of crowns with complete metal coping was found to be higher than the reduced metal coping. These finding were similar to the studies conducted by O'Boyle et al. [4] and Ulusoy and Toksavul [2]. Whereas, Gardner et al. stated that fracture strength of porcelain facial margin was higher than porcelain fused to metal margin in collarless metal ceramic crowns [1].

Shoulder bevel marginal configuration was also being evaluated with different framework reduction. It was seen that the difference between the fracture strength of crowns with 1 mm and 1.5 mm metal framework reduction for shoulder bevel margin group was statistically insignificant. The fracture strength of these collarless metal ceramic crowns with shoulder bevel margin was much more than the normal biting force encountered in the oral cavity. But the fracture strength of complete metal coping was slightly higher than the reduced metal coping. These finding were in co-relation with the study conducted by Lehner et al. [11]. The mean fracture strength of the specimens in this study with different marginal configuration and different framework reductions, was found to be much more than the biting forces cited by various researchers [12–14]. Therefore, it can be documented that the catastrophic marginal failure with either type of margin has not been an area of concern during normal function. It also suggested that the porcelain margin will probably survive functional loads encountered in the oral cavity. Though the study had few limitations in the form of possibility of porcelain seepage occurring under the metal coping cannot be eliminated [1].

## 5. Conclusion

 It can be concluded from this study that ceramo-metal crowns with shoulder margin fractured under higher forces than those with shoulder bevel margin, irrespective of the framework reduction. The fracture strength of crowns with 0.5 mm and 1 mm metal framework reductions for shoulder margin group was nearly the same. The difference between the fracture strength of crowns with 1 mm and 1.5 mm metal framework reduction for shoulder bevel margin group was found to be statistically insignificant. The fracture strength for the crowns with complete metal coping is higher than crowns with reduced metal coping. As the metal reduction increased, the fracture resistance decreased. Therefore, it can be suggested that collarless metal ceramic crowns with either shoulder margin or shoulder bevel margin up to 1.5 mm of metal framework reduction may be advocated in the clinical practice successfully. These newer restorations are durable and also help to impart better esthetics.

## Figures and Tables

**Table 1:** Table showing the fracture strength of the various samples in newtons with standard deviation (SD) and standard error (SE) in mean.

Groups	Subgroup	Mean fracture strength	SD	SE
		(In newtons)		
A	A1	1688.4	177.408	246.24
	A2	893.8	113.6	157.67
	A3	871.2	114.82	159.37
	A4	637	90.939	126.22

B	B1	935.6	150.868	209.40
	B2	732.2	117.824	163.53
	B3	419.2	54.817	76.08
	B4	407.4	50.949	70.71
